# Trends in postgraduate medical education in Iran

**DOI:** 10.1186/1472-6963-14-S2-P124

**Published:** 2014-07-07

**Authors:** Shima Tabatabai, Seyed Amir Mohsen Ziaee

**Affiliations:** 1 School of Medical Education, Shahid Beheshti University of Medical Sciences, Tehran, Iran; 2 Shahid Labbafinejad Medical Center, Shahid Beheshti University of Medical Sciences, Tehran, Iran

## Background

Iran is designing an evidence-based plan for promoting postgraduate medical education and efficient distribution of health professional human resources. An important part of this program is foresight studies for post graduate medical education considering the important trends affecting the future of health status and postgraduate medical education in Iran. In this article, we clarify such trends in Iran.

## Methods

For this study, we used a systematic review of current evidence about the mega trends affecting the future of medical education. Also we gathered opinions of key stakeholders in expert panels.

## Results

Following trends identified as affecting the health system and post graduate medical education in Iran: demographic changes; epidemiologic transition; physician work patterns changes, female specialists number growth, changes in patients’ expectations of health services; growth in information and communication technologies; new advances in diagnostic and therapeutic technologies.

## Conclusions

The present study found that trends affecting postgraduate medical education in Iran. In the near future, medical education in Iran will need to undergo major changes. When planning for these changes, decision-makers should consider the various trends that affect education.

